# Optimization of Variable-Temperature Pressure-Difference Puffing Drying Process for Persimmon Chips Using Response Surface Methodology

**DOI:** 10.3390/foods13233830

**Published:** 2024-11-27

**Authors:** Xiaoxian Tang, Zhaokun Xian, Yan Liu, Aiqing Ren, Hanying Tan, Yuyan Pan, Zhenhua Duan

**Affiliations:** 1 Guangxi Key Laboratory of Health Care Food Science and Technology, Hezhou University, Hezhou 542899, China; tangxiaoxian2016@163.com (X.T.); liuyan130832@126.com (Y.L.); renaiqing@126.com (A.R.); thythy2014@163.com (H.T.); pyy19950116@163.com (Y.P.); 2 College of Mechanical and Control Engineering, Guilin University of Technology, Guilin 541006, China; zhaokunxian@st.gxu.edu.cn

**Keywords:** pressure puffing, process optimization, crispness, porous properties, processing method

## Abstract

This study focused on persimmons and applied variable-temperature pressure-differential puffing drying to produce persimmon chips. The effects of puffing pressure, holding time, drying temperature, and duration on moisture content, crispness, and sensory scores were examined. The optimal parameters determined via response surface methodology were a pressure differential of 0.46 MPa, holding time of 10 min, drying temperature of 94 °C, and drying time of 92 min, achieving a moisture content of 3.63%, crispness of 362.83 g, and sensory score of 90.8. Microstructural and porosity analysis showed that this method predominantly produced chips with large pores and enhanced pore volume (0.2949 cm^3^/g), porosity (30.16%), and average pore diameter (194.0 nm). Compared to microwave drying, vacuum microwave drying, and vacuum frying, the pore volume of persimmon chips processed using pressure-differential puffing drying increased by 57.7% to 237.8%, the porosity improved by 57.2% to 237.8%, and the average pore diameter grew by 82.2% to 660.8%. Notably, the differences were most pronounced when compared to vacuum frying, with increases of 237.8%, 237.8%, and 660.8%, respectively. These results indicate that pressure-differential puffing drying is superior in creating loose structures and achieving high-quality persimmon chips, making it the preferred processing method.

## 1. Introduction

The persimmon belongs to the genus *Diospyros* within the family *Ebenaceae*, which is a dicotyledonous plant [1,2]. Widely cultivated across Asian countries, persimmons are particularly prevalent in China, South Korea, Japan, and India, with China having the most extensive cultivation and distribution [3]. Due to its significant commercial value, persimmon cultivation has expanded to Europe and North America [4]. Persimmons are rich in vitamins, proteins, tannins, minerals, anthocyanins, and phenolic compounds, which confer strong antioxidant properties as well as high nutritional, medicinal, and economic value [5]. Persimmon varieties can be categorized by taste into sweet persimmons and astringent persimmons, with the astringent taste primarily derived from soluble tannins. As a highly seasonal fruit, persimmons have a concentrated ripening and harvest period. Fresh persimmons contain over 80% water, making them susceptible to rapid spoilage and difficult to store, resulting in a short shelf life [6,7]. Delays in the sale or processing of fresh fruit can lead to significant economic losses for farmers due to spoilage and decay.

Drying plays a crucial role in quality control and reducing post-harvest losses of agricultural products during storage, widely used in the food industry [8]. By effectively removing moisture from materials, drying reduces nutrient loss, inhibits microbial growth, and extends shelf life, making it a common method for food preservation and processing. Some studies have already examined the drying of persimmons. Senadeera et al. found low temperature, long-duration, hot-air drying of persimmon slices can cause browning reactions [9]. Jia et al. reported the signification of bioactive substances after hot-air drying due to conditions that favor browning reactions, resulting in notable color degradation in the final product [10]. Sheng et al. observed that high-temperature drying significantly affects the drying characteristics and quality of persimmon slices, with a substantial loss of total phenolics [11]. In convection drying, prolonged contact with hot air led to a loss of both nutritional and sensory quality in dried products [12]. Vacuum-fried persimmon slices exhibited effects and a porous structure, with a smooth oil layer on the surface; however, the high oil content could negatively impact health [13]. According to Çelen S et al., high microwave drying of persimmon led to uneven microwave energy distribution, which could induce browning and compromise the color quality [6]. Tan et al. found that during intermittent microwave-drying of persimmons, the slices exhibited varying browning reactions, with the core region being particularly prone to charring or carbonization [14]. Freeze-drying (FD) uses sublimation to produce high-quality products with a long shelf life and unique texture [15,16]. Zhao et al. discovered that freeze-drying retains higher levels of bioactive compound slices compared to air drying [17]. However, the high energy consumption and cost of this technology limit its use in persimmon drying applications.

Variable-temperature pressure-difference puffing drying is an innovative puffing technology that uses rapid changes in temperature and pressure to act on the material, causing the internal liquid to instantly vaporize and dehydrate. This process resulted in a porous and loose structure due to the expansion forces exerted on the material, producing fruit and vegetable chips with good color, flavor, and a crispy texture [18]. Fruit and vegetable chips were a popular snack with a crispy texture and appealing taste, becoming increasingly popular among young consumers and a growing segment in the food industry [19]. As is well known, products with porous structures possess unique textures and rehydration capabilities, with porosity playing a crucial role in determining food quality [20]. Currently, research on variable-temperature pressure-difference puffing drying technology has been conducted on fruits and vegetables [20,21], edible fungi [22], and insects [23], demonstrating promising applications in food processing. However, there are few reports specifically on persimmons processed with this method. In this study, persimmons were used as the raw material to investigate the effects of variable-temperature pressure-difference puffing drying (puffing pressure difference, drying temperature, and drying time) on key indicators, such as moisture content, crispness, and sensory evaluation scores of persimmon chips. Building on single-factor experiments, the drying process was optimized using response surface methodology to obtain the best process parameters. Additionally, a comparative analysis of the microstructure and porosity characteristics of persimmon chips produced through pressure-difference puffing drying, microwave drying, vacuum microwave drying, and vacuum frying was conducted to provide theoretical reference and technical support for persimmon chip processing.

## 2. Materials and Methods

### 2.1. Materials and Instruments

The persimmons used in this study were astringent persimmons from Gongcheng, Guangxi, purchased from the Taixing Supermarket in Hezhou, Guangxi. The equipment used includes a ZWH-KFY-BT heat pump dryer (Weierxin Industrial Co., Ltd., Shantou, China), a KAAE pressure-difference puffing dryer (Kaiyi Intelligent Technology Co., Ltd., Wuxi, China), a TA.XT PLUS texture analyzer (Stable Micro Systems Ltd., Godalming, UK), a CR-400 colorimeter (Konica Minolta, Tokyo, Japan), a BSA 124 S analytical balance (Sartorius, Göttingen, Germany), an MX-50 moisture analyzer (A&D, Tokyo, Japan), a QUANTA 250 field emission scanning electron microscope (FEI, Hillsboro, OR, USA), an Autopore 9620 mercury intrusion porosimeter (Micromeritics, Atlanta, GA, USA), and a MC1000 Hitachi Ion Sputter Coater, manufactured by Hitachi High Technologies Co., Ltd (Tokyo, Japan).

### 2.2. Experiment and Method

#### 2.2.1. Processing Workflow for Persimmon Chips

Fresh Persimmons → Washing → Peeling and Stem Removal → Slicing → Heat Pump Pre-Drying → Moisture Equalization → Variable-Temperature Pressure-Difference Puffing Drying → Cooling → Packaging → Final Product.

Key Steps: Fresh persimmons were selected according to the national standard of the People’s Republic of China “GB/T 20453-2022 Quality grades of products for persimmon” [24], with a flat and round shape, a single fruit weight of 170 g, an average soluble solid content 21%, fruit flesh hardness >6 kg/cm^2^, orange-yellow flesh, and no mechanical damage. Wash the persimmons, remove the peel and stem, and cut them into horizontal slices with a thickness of 3 mm and a diameter of (85 ± 1) mm. Weigh 500 g of slices and place them in a heat pump dryer for pre-drying (temperature 65 °C, wind speed 4 m/s) until the moisture content is reduced to around 25%. Transfer the pre-dried slices into a resealable bag and allow them to equalize moisture at 4 °C for 12 h. Finally, dry the moisture-equalized persimmon slices using a variable-temperature pressure-difference puffing dryer.

#### 2.2.2. Single Factor Experiment

Effect of puffing pressure difference on persimmon chips. Under conditions of a preheating temperature of 80 °C, a holding time of 15 min, a puffing time of 10 min, a drying temperature of 90 °C, and a drying time of 100 min, the impact of puffing pressure differences (0.35 MPa, 0.40 MPa, 0.45 MPa, 0.50 MPa, and 0.55 MPa) on the moisture content, crispness, and sensory evaluation scores of persimmon chips was examined.

Effect of holding time on persimmon chips. Under conditions of a preheating temperature of 80 °C, a puffing pressure difference of 0.45 MPa, a puffing time of 10 min, a drying temperature of 90 °C, and a drying time of 100 min, the influence of holding times of 5 min, 10 min, 15 min, 20 min, and 25 min on the moisture content, crispness, and sensory evaluation scores of persimmon chips was investigated.

Effect of drying temperature on persimmon chips. Under conditions of a preheating temperature of 80 °C, a puffing pressure difference of 0.45 MPa, a holding time of 10 min, a puffing time of 10 min, and a drying time of 100 min, the impact of drying temperatures (80 °C, 85 °C, 90 °C, 95 °C, and 100 °C) on the moisture content, crispness, and sensory evaluation scores of persimmon chips was examined.

The effect of drying time on persimmon chips was investigated under the following conditions: preheating temperature of 80 °C, expansion pressure difference of 0.45 MPa, pressure-holding time of 10 min, expansion time of 10 min, and drying temperature of 95 °C. Drying times of 80 min, 90 min, 100 min, 110 min, and 120 min were evaluated for their impact on the moisture content, crispness, and sensory scores of persimmon chips.

#### 2.2.3. Experimental Design of Response Surface Optimization

Based on the results of the single-factor experiments, the Box–Behnken experimental design principle was applied. The independent variables selected were pressure difference (X_1_), drying temperature (X_2_), and drying time (X_3_). The response variables for the experimental design were the moisture content of the persimmon chips (Y_1_), crispness (Y_2_), and sensory evaluation scores (Y_3_). The factors and levels for the response surface experimental design are presented in Table 1.

#### 2.2.4. Verification Experiment

Validation experiment. To verify the reliability and stability of the response surface optimization results, three repeated experiments were conducted using the optimal conditions for pressure-difference puffing drying of persimmon chips obtained from the response surface optimization. The results were averaged to confirm the consistency of the optimized conditions.

#### 2.2.5. Moisture Content Determination

The moisture content of the sample was primarily determined by measuring its dry basis moisture content, which was calculated using Equation (1) [25].

(1) $$M_{t}=\frac{m_{t}-m_{0}}{m_{0}}\times 100$$

where *M_t_* is the moisture content of persimmon chips at time t during variable-temperature pressure-difference puffing drying, expressed as a percentage, %. *m_t_* is the mass of the persimmon chips at time t during drying, g. *m*_0_ is the absolute dry mass of the persimmon chips, g.

#### 2.2.6. Crispness Measurement

The crispness of the persimmon chips was measured using a TA.XT PLUS texture analyzer (Stable Micro Systems Ltd., Godalming, UK). The sample was placed on the testing platform, and a P2 probe was used with a test distance of 2 mm, a trigger force of 5.0 g, and a probe speed of 1.0 mm/s. Crispness was indicated by the first distinct peak in the pressure graph as the probe initially compresses the sample. A lower crispness value represented better crispness [23].

#### 2.2.7. Sensory Evaluation

A panel of 20 food professionals trained in sensory evaluation assessed the persimmon chip samples. The sensory evaluation criteria were outlined in Table 2, focusing on four sensory attributes: color, appearance, texture, and aroma. Each sample was scored based on these attributes, with the overall score calculated on a 100-point scale. The sensory evaluation score for each sample was recorded as the average of the panelists’ scores.

#### 2.2.8. Microstructure Analysis

The microstructure of the samples was analyzed using a QUANTA250 (FEI, Hillsboro, OR, USA) field emission scanning electron microscope, which provided comprehensive information on pore structure morphology. Small sample pieces were cut with a blade (5 mm × 5mm × 0.25mm), and the surfaces were gold-coated using a Hitachi MC1000 (Tokyo, Japan) ion sputtering device under the following conditions: 10 mA current for 80 s. The gold-sprayed sample was placed in the scanning electron microscope sample chamber, with the voltage (HV) set to 20.0 kV and the working distance (WD) set to 8 mm. Scanning and analysis were then performed at magnifications of 100× (scale = 1 mm), 500× (scale = 200 µm), and 1000× (scale = 10 µm).

#### 2.2.9. Measurement of Pore Characteristics

The mercury intrusion method was an effective technique for assessing the pore size distribution, porosity, average pore diameter, total pore area, and apparent density of porous materials. In this method, the amount of mercury intruded was considered equal to the total pore volume of the sample. The pore size distribution of the persimmon chip samples was measured using mercury intrusion porosimetry, with a pressure range of 0.0034 MPa to 429.9 MPa and pore size range of 2.90 nm to 361,727.08 nm. The relationship between sample pressure and pore diameter was described by the Washburn equation (Equation (2)) [26], which illustrates the linear correlation between the size of pores (corresponding to intruded volume) and the applied mercury pressure. The pore size distribution curve reflects the equivalent volume of Hg intruded (cm^3^/g) at various pore sizes [27]. Bulk density was calculated as the ratio of sample mass to total volume, representing the mass per unit volume of the sample in g/cm^3^.

(2) $$P=\frac{4\gamma_{Hg}\cos\theta }{\emptyset }$$

where P is the external pressure applied in the vacuum chamber, in MPa; *γ_Hg_* is the surface tension of mercury; 0.48 N/m is the contact angle of mercury (140°); and Ø is the diameter of the pore, in µm.

#### 2.2.10. Other Processing Methods for Persimmon Chips

For each group, 500 g of fresh persimmon slices (a thickness of 3 mm and a diameter of (85 ± 1) mm) were used for different processing methods.

(1) Microwave drying. The intermittent microwave drying method was employed, with a power setting of 140 W. The process involved a cycle of 1 min of microwave heating followed by a 0.5min pause, continuing until the moisture content of the material was reduced to ≤4% (wet basis).

(2) Vacuum microwave drying. The vacuum was maintained at 0.095 MPa with a microwave power of 300 W. The drying process was terminated when the moisture content of the material reached ≤4% (wet basis).

(3) Vacuum frying. The vacuum was set at 0.090 MPa, and frying was conducted at a temperature of 95 °C. The de-oiling speed was set to 300 r/min, with a frying duration of 6 min followed by a de-oiling period of 3 min. The process was concluded when the moisture content of the material was ≤4% (wet basis).

### 2.3. Data Processing

Each group of experiments was repeated three times, and the experimental results were processed using Excel 2019 software. The response surface design and data analysis were conducted using Design-Expert 8.0.6 software. Data visualization and statistical analysis were performed with Origin 2018 and SPSS 26.0 software.

## 3. Results and Discussion

### 3.1. Single-Factor Experimental Results

#### 3.1.1. Effect of Expansion Pressure Difference on the Quality of Persimmon Chips

**Figure 1:**
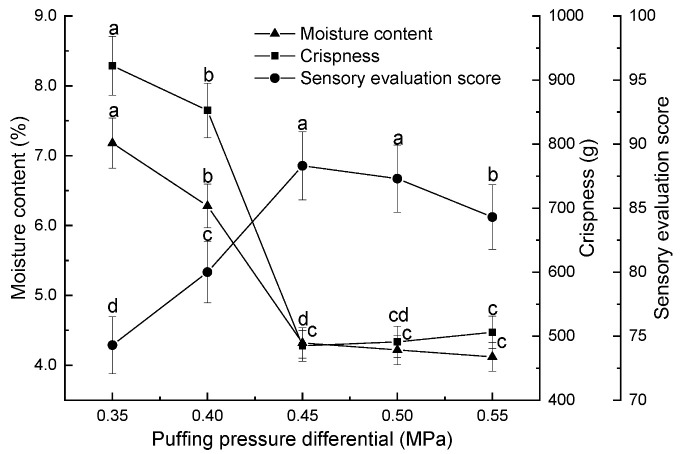
Effect of expansion pressure differential on moisture content, crispness, and sensory scores of persimmon chips (note: different lowercase letters indicate significant differences at *p* < 0.05; the same applies to the following figures).

The expansion pressure difference refers to the difference between the pressure in the expansion chamber and the vacuum chamber before the material expands. It was an important parameter in variable temperature pressure differential expansion, which causes the moisture in the material, possessing a certain amount of energy, to instantaneously transition from the liquid phase to the gaseous phase. As shown in Figure 1, with an increase in the expansion pressure difference, the moisture content of the persimmon chips decreased and the crispness value initially decreased rapidly and then increased slowly, while the sensory evaluation score first increased and then decreased. In the range from 0.35 to 0.45 MPa, the moisture content and crispness values significantly decreased, but the sensory score did not improve. When the expansion pressure difference reached or exceeded 0.45 MPa, there was no significant difference in moisture content (*p* > 0.05), with moisture levels ranging from 4.12% to 4.32%. At this pressure difference, the crispness value was minimized at 484.91 g, indicating the best crispness and texture, characterized by a uniform, bright golden color and a strong persimmon aroma, resulting in the highest sensory score of 88.3. When the pressure difference exceeds 0.45 MPa, the crispness value gradually increases, leading to a slight reduction in crispness and texture, as well as a decrease in the sensory score. Therefore, selecting expansion pressure differences of 0.40 MPa, 0.45 MPa, and 0.50 MPa for response optimization experiments was advisable.

#### 3.1.2. Effect of Holding Time on the Quality of Persimmon Chips

**Figure 2:**
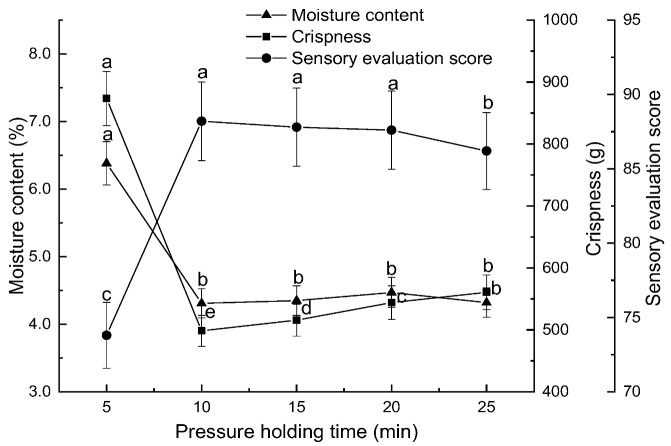
Effect of pressure holding time on moisture content, crispness, and sensory scores of persimmon chips (note: different lowercase letters indicate significant differences at *p* < 0.05; the same applies to the following figures).

During the process of pressure differential expansion drying, the application of pressure over time can lead to phase changes, molecular structure alterations, chemical reactions, and even the rearrangement of molecular sequences. These changes do not occur instantaneously but evolve over time. Given a constant pressure, the holding time represents the duration over which these macroscopic changes in the material were achieved [28]. As shown in Figure 2, with an increase in holding time, the moisture content of persimmon chips decreased and eventually stabilized. The crispness value initially decreased and then increased slightly, while the sensory score initially rose and then stabilized. At a holding time of 10 min, the moisture content (4.31%) and crispness value (498.5 g) reach their lowest levels, yielding persimmon chips with a bright, uniform color; intact shape; excellent crispness; and a strong persimmon aroma. At this point, the sensory score reached its peak at 88.2. When the holding time exceeded 10 min, the moisture content remained within 4.31% to 4.47%, and while crispness slightly increased and crispness diminished somewhat, there was minimal impact on the overall sensory quality of the persimmon chips, resulting in only minor fluctuations in the sensory score from the maximum value. Therefore, a holding time of 10 min was selected for subsequent experiments.

#### 3.1.3. Effect of Drying Temperature on the Quality of Persimmon Chips

**Figure 3:**
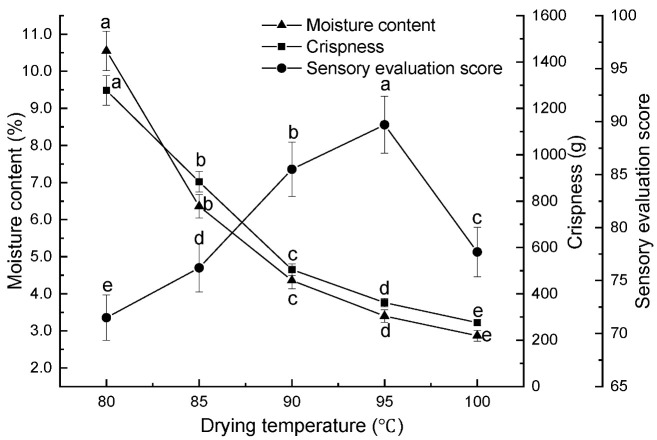
Effect of drying temperature on moisture content, crispness, and sensory scores of persimmon chips (note: different lowercase letters indicate significant differences at *p* < 0.05; the same applies to the following figures).

As shown in Figure 3, both moisture content and crispness values of the persimmon chips decreased with increasing drying temperature. At 100 °C, the moisture content (2.87%) and crispness value (275.6 g) reached their lowest points, resulting in improved crispness. However, the sensory score initially rose and then decreased; it peaked at 89.7 when the drying temperature was 95 °C. At this point, the persimmon chips displayed a bright yellow color, with a crisp texture and strong persimmon aroma. When the drying temperature exceeded 95 °C, although the crispness of the chips was slightly better compared to lower temperatures, the overall color became darker, shifting to a deeper yellow with a slight caramelized flavor, which lowers the sensory score. This was due to the high drying temperature causing the surface moisture to evaporate at a slower rate than the internal moisture diffusion, leading to uneven heat distribution and surface browning. As a result, the color darkened noticeably, and the texture deteriorated, which negatively impacts the sensory appeal. This observation was consistent with findings by Liu et al. [29]. on the vacuum differential expansion-drying of mushroom chips. Based on the above results, drying temperatures of 90 °C, 95 °C, and 100 °C would be selected for further response optimization experiments.

#### 3.1.4. Effect of Drying Time on the Quality of Persimmon Chips

**Figure 4:**
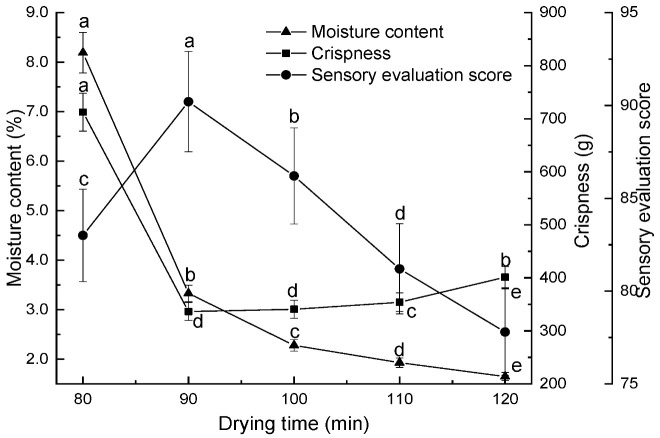
Effect of drying time on moisture content, crispness, and sensory scores of persimmon chips (note: different lowercase letters indicate significant differences at *p* < 0.05; the same applies to the following figures).

Drying time refers to the duration over which moisture was further removed from the material in a vacuum state at a set drying temperature after expansion. As illustrated in Figure 4, with other conditions held constant, the moisture content of persimmon chips decreased as drying time increased, while crispness first decreased and then increased. Similarly, the sensory score initially rose but then declined. At 80 min, drying was insufficient, leaving a considerable amount of moisture (8.19%) in the persimmon chips. The crispness value was high (712.3 g), indicating weaker crispness, with lower brightness and yellowness, resulting in a lower sensory score. When the drying time was extended to 90 min, additional moisture was removed, reducing the moisture content to 3.33%. This facilitated the formation and solidification of the chips’ porous structure. At this point, the crispness value drops to 336.6 g, resulting in a desirable crisp texture and strong persimmon aroma, along with a bright yellow color, achieving a sensory score of 90.2. If the drying time exceeded 90 min, the moisture content further decreased, but crispness values increased, diminishing the crispness. Prolonged drying causes sugars to migrate to the surface, forming a hard film and increasing hardness, which negatively impacts crispness. Additionally, the excess heat energy from extended drying could cause charring, masking the persimmon aroma with a caramelized scent. The color also darkened, leading to a decline in sensory score. Based on these results, drying times of 80 min, 90 min, and 100 min would be selected for subsequent response optimization experiments.

### 3.2. Results of Response Surface Analysis

#### 3.2.1. Response Surface Experimental Design and Results

Based on the results from single-factor experiments, a holding time of 10 min was set for the response surface optimization. The factors selected as independent variables were pressure difference (X_1_), drying temperature (X_2_), and drying time (X_3_), while the response values were moisture content (Y_1_), crispness (Y_2_), and sensory score (Y_3_) of the persimmon chips. Response surface methodology (RSM) was employed to optimize the variable-temperature differential pressure drying parameters for persimmon chips. The experimental design and results are presented in Table 3.

Using Design-Expert 8.0.6 software, multiple regression fitting was conducted on the optimization experiment results from Table 3. This analysis produced quadratic polynomial regression models that describe the relationships between moisture content (Y_1_), crispness (Y_2_), and sensory score (Y_3_) with the factors of pressure difference (X_1_), drying temperature (X_2_), and drying time (X_3_).

Y_1_ = 3.71 + 0.25X_1_ − 1.56X_2_ − 1.36X_3_ − 0.005X_1_X_2_ + 0.21X_1_X_3_ + 1.24X_2_X_3_ + 0.16X_1_^2^ − 0.32X_2_^2^ + 0.77X_3_^2^ (3)

Y_2_ = 372.85 − 43.37X_1_ − 149.52X_2_ − 155.67X_3_ + 14.47X_1_X_2_ + 29.42X_1_X_3_ + 133.54X_2_X_3_ + 56.24X_1_^2^ + 16.37X_2_^2^ + 86.34X_3_^2^ (4)

Y_3_ = 90.10 + 0.69X_1_ + 1.31X_2_ + 1.88X_3_ + 0.13X_1_X_3_ − 3.88X_2_X_3_ − 3.18X_1_^2^ − 2.68X_2_^2^ − 2.80X_3_^2^ (5)

After conducting variance analysis on the regression equations, the variance analysis results for each response value are shown in Tables S1–S3 (Supplementary Materials). The *p*-values for the three response models—moisture content (Y_1_), crispness (Y_2_), and sensory score (Y_3_)—all reached a highly significant level (*p* < 0.01). The lack-of-fit p-values for Y_1_ moisture content and Y_2_ crispness were <0.0001, indicating a highly significant lack of fit (*p* < 0.01). For Y_3_ sensory score, the lack-of-fit *p*-value was 0.0348, showing a significant difference (*p* < 0.05). In the moisture content model, X_2_, X_3_, and the interaction term X_2_X_3_ had highly significant effects on the response value (*p* < 0.01), while the quadratic term X_3_^2^ was significant. However, X_1_, X_1_X_2_, X_1_X_3_, X_1_^2^, and X_3_^2^ did not significantly affect the response (*p* > 0.05). For the crispness model, X_1_, X_2_, X_3_, X_2_X_3_, X_1_^2^, and X_3_^2^ had highly significant impacts on the response (*p* < 0.01), while X_1_X_2_, X_1_X_3_, and X_2_^2^ did not significantly affect the response (*p* > 0.05). In the sensory score model, X_2_, X_3_, X_2_X_3_, X_1_^2^, X_2_^2^, and X_3_^2^ had highly significant effects on the response (*p* < 0.01), while X_1_, X_1_X_2_, and X_1_X_3_ did not significantly affect the response (*p* > 0.05). The determination coefficient R^2^ values for the three models were 0.9692, 0.9876, and 0.9780, with adjusted R^2^ (R^2^_Adj_) values of 0.9296, 0.9718, and 0.9500, respectively. Since both R^2^ and R^2^_Adj_ were greater than 0.9, the models demonstrate high consistency between the tested and predicted values, indicating good correlation and the ability to accurately predict the evaluation indicators of moisture content, crispness, and sensory score for the persimmon chips. According to Tables S1–S3 (Supplementary Materials), the order of influence of X_1_ pressure difference, X_2_ drying temperature, and X_3_ drying time on moisture content, crispness, and sensory score were as follows: X_2_ > X_3_ > X_1_ for moisture content, X_3_ > X_2_ > X_1_ for crispness, and X_1_ > X_2_ > X_3_ for sensory score. The order of interaction effects between the factors was X_2_X_3_ > X_1_X_3_ > X_1_X_2_ for moisture content, X_1_X_2_ > X_2_X_3_ > X_1_X_3_ for crispness, and X_1_X_3_ > X_2_X_3_ > X_1_X_2_ for sensory score.

#### 3.2.2. Response Surface Curve Analysis

**Figure 5:**
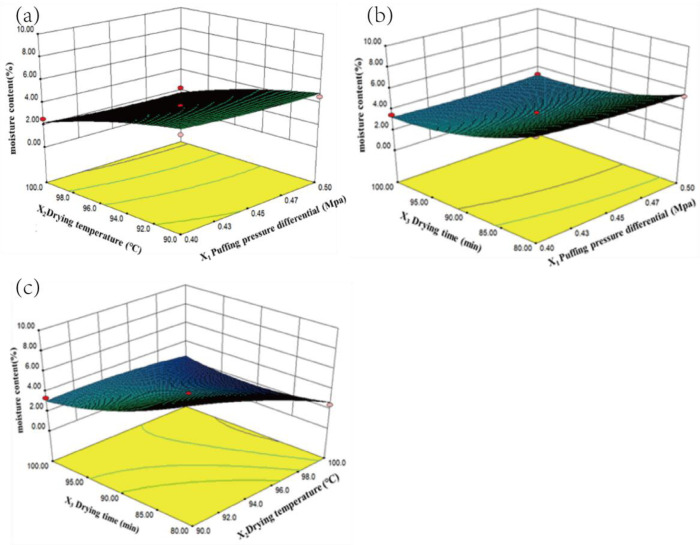
Response surface plot showing the interactive effects of factors on moisture content ((**a**): X_3_ = 90 min, (**b**): X_2_ =90 °C, and (**c**): X_1_ = 0.45 MPa).

In Figure 5, the moisture content (Y_1_) of the persimmon chips decreased as the pressure difference (X_1_), drying temperature (X_2_), and drying time (X_3_) increased. The decrease in Y_1_ was gradual with an increase in X_1_, and the interactions between X_1_ and X_2_ and between X_1_ and X_3_ were not significant. In contrast, Y_1_ decreased rapidly with increasing X_2_ and X_3_, with steep response surface slopes for both X_2_ and X_3_, indicating a significant interaction between these variables, which facilitated accelerated moisture loss in the samples. This result aligns with the findings of Yang Hui et al. in their study on drying cantaloupe [8].

**Figure 6:**
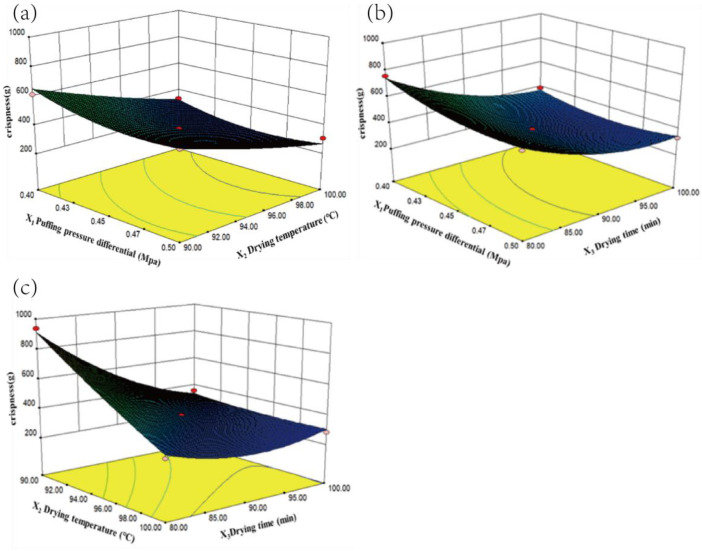
Response surface plot showing the interactive effects of factors on crispness ((**a**): X_3_ = 90 min, (**b**): X_2_ = 90 °C, and (**c**): X_1_ = 0.45MPa.)

As shown in Figure 6, the crispness value (Y_2_) decreased rapidly with increasing X_2_ and X_3_, indicating an improvement in crispness. Similarly, within the range of 0.40 MPa–0.50 MPa, the effect of X_1_ (pressure difference) on crispness was relatively minor. However, X_2_ and X_3_ had a direct impact on the final crispness of the samples, with X_1_ also serving as an important parameter. This was because, as the drying temperature increased, the moisture in the persimmon chips rapidly vaporized, and the sudden release of moisture under expansion forces led to a more porous structure, enhancing crispness [30]. Additionally, sufficient drying time contributed to stabilizing the texture quality of the samples.

**Figure 7:**
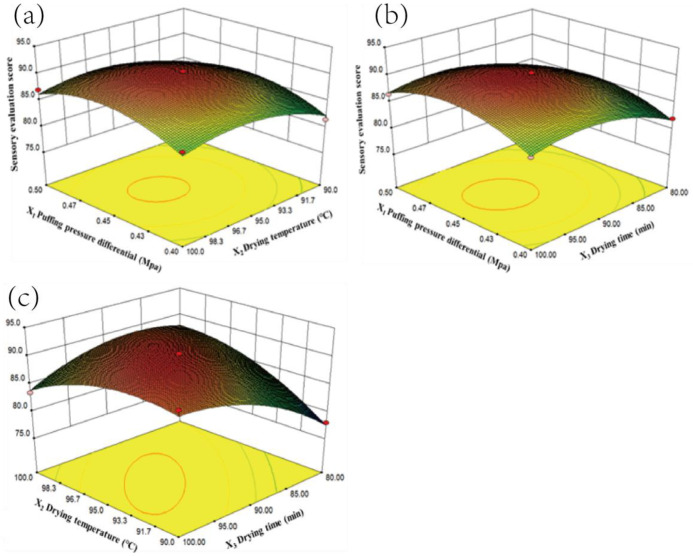
Response surface plot of the interaction effects of various factors on sensory scores ((**a**): X_3_ = 90 min, (**b**):X_2_ = 90 °C, and (**c**): X_1_ = 0.45 MPa.)

As shown in Figure 7a, when X_2_ was at a low level, the sensory score (Y_3_) increased as X_2_ rose, reaching its highest value at the central level of X_2_, with X_1_ having a relatively minor effect. This was because higher drying temperatures provided energy for moisture vaporization, enhanced gas expansion, and created a loose, porous structure, which improved the crispness and palatability of the persimmon chips and intensified their aroma, thereby increasing the sensory score. As seen in Figure 7b, when X_2_ was held at its central level, the sensory score (Y_3_) increased with rising X_1_ and X_3_. However, when X_3_ exceeded 90 min, the sensory score began to decrease; when X_1_ surpassed 0.45 MPa, the sensory score continued to rise. According to Figure 7c, with X_1_ set at its central level, the sensory score (Y_3_) initially increased and then decreased with rising X_2_ and X_3_. At lower levels of X_2_, the sensory score rose as X_3_ was extended; at higher levels of X_2_, the sensory score quickly declined with longer X_3_. The highest sensory score was achieved when X_3_ was at its central level, with a strong influence from drying temperature; when X_3_ deviated from this level, the sensory score gradually decreased. This decline occurred because excessive drying temperature and prolonged drying time caused over-dehydration, structural fractures, and a decrease in texture quality. Additionally, non-enzymatic browning reactions, such as the Maillard reaction, caramelization, and oxidation of phenolic compounds, produced brown pigments that darkened and yellowed the color of the persimmon chips. Furthermore, intensified protein denaturation impaired the puffing effect, ultimately lowering the sensory score. This trend was consistent with findings by Song et al. in their study on vacuum-puffed dried yellow peach chips [31].

#### 3.2.3. Process Prediction and Validation

To determine the optimal pressure-differential puffing drying process for persimmon chips, experimental data were analyzed using Design-Expert 8.0.6 software under a holding time of 10 min. The software suggested optimal conditions of a pressure differential of 0.46 MPa, a drying temperature of 93.67 °C, and a drying time of 92.41 min. The model predicted that, under these conditions, the moisture content would be 3.71%, the crispness would measure 366.67 g, and the sensory score would be 90.1. Adjusted based on practical considerations, the final conditions were set as a pressure differential of 0.46 MPa, drying temperature of 94 °C, and drying time of 92 min.

The optimal conditions were tested in triplicate. The average experimental results yielded a moisture content of 3.69%, crispness of 362.83 g, and a sensory score of 90.8. The experimental results were 0.54%, 1.01%, and 0.77% higher than the predicted values, respectively. This demonstrates good reproducibility, indicating that the model can accurately predict the actual conditions. The response surface optimization for the pressure-differential puffing drying parameters of persimmon chips proved to be accurate and has practical application value.

### 3.3. Comparison of the Microstructure of Persimmon Chips Processed by Different Methods

**Figure 8:**
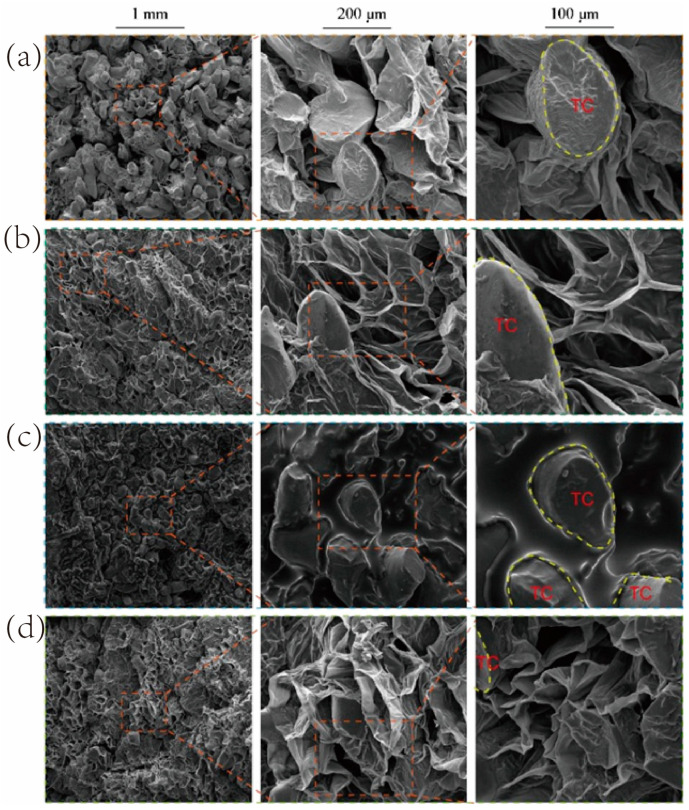
Microscopic structure images of persimmon chips processed by different methods. (**a**) Microwave drying, (**b**) vacuum microwave drying, (**c**) vacuum frying, (**d**) pressure differential puffing drying; TC denotes tannin cells.

As shown in Figure 8, the microstructures of persimmon chips processed by different methods revealed significant differences. All samples exhibited good puffing effects and contained solid, irregularly shaped tannin cells (TC) resembling elliptical cross-sections, similar to the tannin cell structures observed in the persimmon ripening study by Tessmer M. A. and colleagues [32]. However, unlike fresh persimmons (shown in Figure S1), the tannin cells in these processed samples appeared to be fewer and exhibited a degree of cell wall distortion. This likely results from thermal processing, as heat can cause tannin degradation or transformation, reducing their numbers [7]. While some tannin cells with warped cell walls remain, the exact mechanism was unclear, potentially relating to tannic acid content.

Microwave drying disrupts the tissue structure, leading to irregularly shaped pores of varying sizes and orientations. This occurs because microwaves penetrate the cells, causing polar molecules like water to vibrate and produce heat, rapidly elevating the temperature inside the material. The rapid evaporation of moisture from inside the material creates numerous pores, while the fast dehydration process causes structural deformation and surface shrinkage [33]. Additionally, uneven heating results in certain areas absorbing excessive microwave energy, breaking some cell structures and forming larger “air pockets”.

In contrast, vacuum microwave drying produces a looser cell framework with a variety of pore sizes and grooves. Compared to microwave drying, the cell walls in vacuum microwave-dried samples appear thinner but more intact. This difference arises because vacuum microwave drying causes rapid internal moisture evaporation, generating internal pressure and expansion forces that create porous structures. The vacuum environment preserves cell walls and membranes, reducing rupture and deformation. The resulting pores also help prevent excessive shrinkage and deformation during drying.

The vacuum-fried samples feature a smooth oil layer on the surface, with pores inside filled with oil. This effect stems from rapid moisture vaporization under low pressure, causing cell expansion and structural breakdown, which increases pore volume and oil absorption [12].

Pressure-differential puffing drying results in loose, porous structures with well-connected pores, indicating good puffing and crispness. This method involves heating the material under pressure, keeping internal moisture in a superheated state. Upon sudden decompression, the rapid phase change forces the superheated liquid to vaporize, creating a porous, loose structure that enhances crispness. Compared to other methods, the pressure-differential puffed samples contain smaller and fewer tannin cells. This observation raises the possibility that pressure-differential puffing may effectively reduce tannin content and astringency in persimmons, warranting further research.

### 3.4. The Impact of Different Processing Methods on Pore Size Distribution in Persimmon Chips

**Figure 9:**
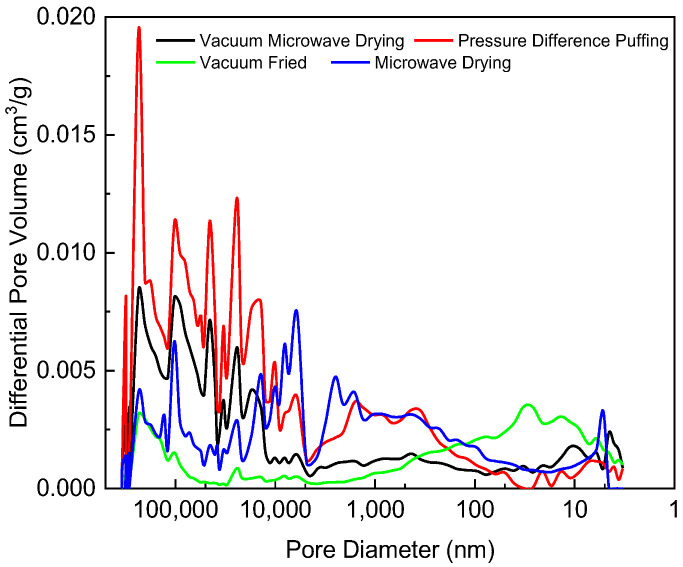
Pore diameter distribution curves of persimmon chips processed by different methods.

Pore size distribution directly affected the texture and mouthfeel of dried fruit and vegetable products. An appropriate pore size distribution could enhance crispness and chewability. In dried fruit and vegetable products, pore size distribution was a complex and crucial characteristic influenced by various factors, including the type of produce, pretreatment conditions, and processing methods. According to the IUPAC classification, pore sizes were categorized as micropores (<2 nm), mesopores (2 nm–50 nm), and macropores (>50 nm) [34]. For persimmon chips processed by the four different methods, pore sizes ranged from 2.90 nm to 361,727.08 nm, primarily in the macropore and mesopore categories. As shown in Figure 9, the pore size distribution curve of vacuum-fried samples exhibits only a broad peak with a lower intensity, indicating a relatively wide range of pore sizes. There was a broad peak around 32 nm and a smaller peak at 233,172 nm, suggesting the presence of a dispersed pore size distribution with a significant amount of mesopores.

In contrast, the pressure-differential puffing, vacuum microwave drying, and microwave drying samples show multiple sharp peaks skewed toward the larger pore size region on the left side of the distribution curve. This indicates that pores of similar sizes were concentrated in several distinct regions, resulting in a non-uniform distribution of macropores. This sharp peak distribution pattern highlights the complexity of the porous structure in dried fruit and vegetable products. The peaks for the pressure-differential puffed sample were significantly higher than those for the other samples, indicating a greater quantity of pores in this size range. A higher peak value corresponds to a larger number of pores of that specific size.

### 3.5. The Impact of Different Processing Methods on the Porosity Characteristics of Persimmon Chips

Porosity characteristics were essential parameters for describing the internal structure of porous media in fruits and vegetables. These characteristics primarily include pore volume, porosity, average pore diameter, total pore surface area, and apparent density. Table 4 indicates that the pore volume, porosity, and average pore diameter of the samples processed using different methods differ significantly (*p* < 0.05). The ranking order for these parameters was pressure-differential puffing drying > microwave drying > vacuum microwave drying > vacuum frying. The pore volume (0.2949 cm^3^/g), porosity (30.16%), and average pore diameter (194.0 nm) of the pressure-differential puffed samples were considerably greater than those of samples produced by the other three methods. The pressure-differential puffed samples exhibit the highest values for porosity, total pore volume, and average pore diameter, which aligns with the loose, well-puffed structure observed in Figure 8. During pressure-differential drying, the rapid vaporization of water in the persimmon chips under overheated conditions creates substantial steam pressure, causing the internal structure to expand and cell walls to burst, forming numerous large pores. This suggests a high degree of puffing. The pore volume, porosity, and average pore diameter of the microwave-dried samples were only slightly lower than those of the pressure-differential puffed samples but higher than those of the other two types. In microwave drying, the rapid vaporization of water creates steam that was unable to diffuse promptly, causing the expanding steam to enlarge the pore structure, resulting in large pore sizes, pore volume, and porosity. The ranking for the total pore surface area was the inverse of the ranking for pore volume and average pore size, with vacuum-fried samples having the largest total pore surface area and pressure-differential puffed samples the smallest. As for apparent density, vacuum microwave drying samples showed slightly lower values, while the other three methods had no significant difference (*p* > 0.05). The pressure-differential puffed samples had the largest pore volume, porosity, and average pore size, yet the smallest total pore surface area. This may be due to the presence of large pores that were more concentrated and well-connected, leading to a smaller total pore surface area. Conversely, vacuum-fried samples predominantly featured mesopores, which were small but numerous, contributing to a smaller total pore volume but a larger total pore surface area.

## 4. Conclusions

This study aimed to investigate the application of differential pressure puffing drying technology in producing high-quality persimmon chips. Using response surface methodology, we identified the optimal processing conditions to achieve low moisture content and improved texture. The experimental results revealed that, compared to other drying techniques, the differential pressure puffing drying method performed exceptionally well in controlling moisture and crispness while significantly enhancing the porosity characteristics of persimmon chips, including increased pore volume, porosity, and average pore size. This method effectively improved the textural structure and sensory quality of the persimmon chips. These findings provide experimental data to support the production and processing of high-quality persimmon chips. However, further research is needed to examine the retention and changes in nutritional components during the differential pressure puffing drying process.

## Figures and Tables

**Table 1:** Factor and level of response surface test design.

Levels	Pressure Difference X_1_	Drying Temperature X_2_	Drying Time X_3_
	(MPa)	(°C)	(min)
−1	0.40	90	80
0	0.45	95	90
1	0.50	100	100

**Table 2:** Sensory evaluation criteria.

Evaluation Criteria	Descriptive Characteristics	Assigned Score
Color: 15 points	Uniform, bright golden yellow	11~15
	Fairly uniform, with a deeper yellow hue	6~10
	Uneven color distribution, with some areas showing darker hue	0~5
Appearance: 15 points	Intact shape, loose structure, uniformly distributed pores	11~15
	Mostly intact shape, loose structure, fairly even pore distribution	6~10
	Incomplete shape, with cracks, curled edges, severe wrinkling, and uneven pores	0~5
Texture: 40 points	Exceptionally crisp, moderate hardness, excellent palatability	31~40
	Fairly crisp, moderate hardness, good palatability	21~30
	Moderate crispness, relatively hard, average palatability	11~20
	Soft, no crispness, poor palatability	0~10
Aroma: 30 points	Strong persimmon aroma, no astringency Noticeable persimmon aroma, no astringency	20~30 13~19
	Faint persimmon aroma, slight astringency	7~12
	Weak persimmon aroma, with strong astringency, bitterness, or off-flavors	0~6

**Table 3:** Design and results of response surface test.

Test	Difference in Pressure X_1_ (MPa)	Drying Temperature X_2_ (°C)	Drying Time X_3_ (min)	Moisture Content Y_1_ (%)	Crispness Y_2_ (g)	Sensory Evaluation Score Y_3_
1	0.4	90	90	4.88	613.41	81.5
2	0.5	90	90	4.55	531.42	83.0
3	0.4	100	90	2.56	330.55	85.5
4	0.5	100	90	2.21	306.44	87.0
5	0.4	95	80	6.42	759.27	82.0
6	0.5	95	80	5.36	580.01	83.0
7	0.4	95	100	3.50	391.79	85.0
8	0.5	95	100	3.28	330.22	86.5
9	0.45	90	80	8.82	938.09	78.0
10	0.45	100	80	2.43	326.85	87.0
11	0.45	90	100	3.41	356.98	90.0
12	0.45	100	100	1.97	279.91	83.5
13	0.45	95	90	3.75	377.65	89.5
14	0.45	95	90	3.73	374.39	90.0
15	0.45	95	90	3.69	370.37	90.5
16	0.45	95	90	3.71	371.54	90.0
17	0.45	95	90	3.69	370.28	90.5

**Table 4:** The porosity characteristics of persimmon chips.

Processing Method	Pore Volume	Porosity	Average Diameter	Total Pore Area	Apparent Density
	(cm^3^/g)	(%)	(nm)	(m^2^/g)	(g/cm^3^)
MD	0.1870 ± 0.007 ^b^	19.19 ± 0.02 ^b^	106.49 ± 1.00 ^b^	6.12 ± 0.09 ^c^	1.471 ± 0.063 ^a^
VMD	0.1628 ± 0.001 ^c^	15.67 ± 0.10 ^c^	67.21 ± 0.45 ^c^	11.12 ± 0.20 ^b^	1.007 ± 0.002 ^b^
VF	0.0873 ± 0.002 ^d^	11.33 ± 0.02 ^d^	25.51 ± 0.35 ^d^	13.68 ± 0.11 ^a^	1.463 ± 0.001 ^a^
PDP	0.2949 ± 0.001 ^a^	30.16 ± 0.03 ^a^	194.02 ± 0.65 ^a^	6.08 ± 0.05 ^c^	1.465 ± 0.001 ^a^

All values were mean ± SD of triplicate measurements. Means within a row with different letters were significantly different (*p* < 0.05).

## Data Availability

The original contributions presented in this study are included in the article/Supplementary Materials, and further inquiries can be directed to the corresponding author.

## Supplementary Materials

The following supporting information can be downloaded at: https://www.mdpi.com/article/10.3390/foods13233830/s1, Table S1 Analysis of variance of regression equation model for moisture content of Persimmon crisps. Table S2. Analysis of variance of regression equation model for the crispness of persimmon crisps. Table S3. Analysis of variance of regression equation model for sensory score of Persimmon Crisp. Figure S1. (a) Tannin cells in fresh persimmon slices ([31]), (b) Tannin cells after pressure-differential puffing drying.
