# Supplementary material for: Optimization of Variable-Temperature Pressure-Difference Puffing Drying Process for Persimmon Chips Using Response Surface Methodology
Source: Foods. 2024 Nov 27;13(23):3830. doi: 10.3390/foods13233830 (PMC11639752; doi:10.3390/foods13233830)
Supplement: Supplementary file 1 [file foods-13-03830-s001.zip › foods-3301449-supplementary.pdf]

Optimization of Variable-Temperature Pressure-Difference Puffing Drying Process for Persimmon Chips Using Response Surface Methodology

Xiaoxian Tang [1,2], Zhaokun Xian [3], Yan Liu [1,2], Aiqing Ren [1,2], Hanying Tan [2], Yuyan Pan [1,2] and Zhenhua Duan [1,2,*]

[1]Guangxi Key Laboratory of Health Care Food Science and Technology (Hezhou University) Hezhou 542899, Guangxi, China; tangxiaoxian2016@163.com

[2]Guangxi Key Laboratory of Health Care Food Science and Technology, Hezhou 542899, China; dzh65@126.com

[3]College of Mechanical and Control Engineering, Guilin University of Technology, Guilin 541006, China; zhaokunxian@st.gxu.edu.cn

*Correspondence: dzh65@126.com

Table S1 Analysis of variance of regression equation model for moisture content of Persimmon crisps.

Source	Sum of Squares	DF	Mean Square	F- Value	P- value
Model	44.01	9	4.897	24.48	0.0002
X_1	0.48	1	0.48	2.40	0.1650
X_2	19.50	1	19.50	97.61	< 0.0001
X_3	14.77	1	14.77	73.93	< 0.0001
X_1X_2	0.00	1	0.00	0.00	0.9828
X_1X_3	0.18	1	0.18	0.88	0.3787
X_2X_3	6.13	1	6.13	30.66	0.0009
X_1^2	0.11	1	0.11	0.53	0.4885
X_2^2	0.44	1	0.44	2.20	0.1814
X_3^2	2.48	1	2.48	12.39	0.0097
Residual	1.40	7	0.12		
Lack of Fit	1.40	3	0.47	684.18	< 0.0001
Pure Error	0.00	4	0.00		
Cor Total	45.41	16			
		R_{Adj}^2=0.9296	R^2=0.9692	R_{Pre}^2= 0.5081	

Table S2. Analysis of variance of regression equation model for the crispness of persimmon crisps.

Source	Sum of Squares	DF	Mean Square	F- Value	P- value
Model	5.13×10^5	9	5.70×10^4	62.37	< 0.0001
X_1	1.50×10^4	1	1.50×104	16.47	0.0048
X_2	1.79×10^5	1	1.79×10^5	195.78	< 0.0001
X_3	1.94×10^5	1	1.94×10^5	212.21	< 0.0001
X_1X_2	8.38×10^2	1	8.38×10^2	0.92	0.3702
X_1X_3	3.46×10^3	1	3.46×10^3	3.79	0.0926
X_2X_3	7.13×10^4	1	7.13×10^4	78.09	< 0.0001
X_1^2	1.33×10^4	1	1.33×10^4	14.58	0.0066
X_2^2	1.13×10^3	1	1.13×10^3	1.24	0.3030
X_3^2	3.13×10^4	1	3.13×10^4	34.28	0.0006
Residual	6.39×10^3	7	9.13×10^2		
Lack of Fit	6.35×10^3	3	2.12×10^3	212.42	< 0.0001
Pure Error	3.99×10	4	9.97		
Cor Total	5.19×10^5	16			
		R_{Adj}^2=0.9718	R^2=0.9876	R_{Pre}^2= 0.804	

Table S3. Analysis of variance of regression equation model for sensory score of Persimmon Crisp.

Source	Sum of Squares	DF	Mean Square	F- Value	P- value
Model	2.24×10^2	9	24.86	34.71	< 0.0001
X_1	3.78	1	3.78	5.28	0.0552
X_2	1.38×10	1	13.78	19.25	0.0032
X_3	2.81×10	1	28.13	39.28	0.0004
X_1X_2	-8.53×10^{-14}	1	0.00	0.00	1.0000
X_1X_3	6.25×10^{-2}	1	0.06	0.09	0.7762
X_2X_3	6.01×10	1	60.06	83.88	< 0.0001
X_1^2	4.24×10	1	42.44	59.27	0.0001
X_2^2	3.01×10	1	30.13	42.08	0.0003
X_3^2	3.30×10	1	33.01	46.10	0.0003
Residual	5.01	7	0.72		
Lack of Fit	4.31	3	1.44	8.21	0.0348
Pure Error	7.00×10^{-1}	4	0.18		
Cor Total	2.29×10^2	16			
		R_{Adj}^2=0.9500	R^2=0.9780	R_{Pre}^2= 0.6936	

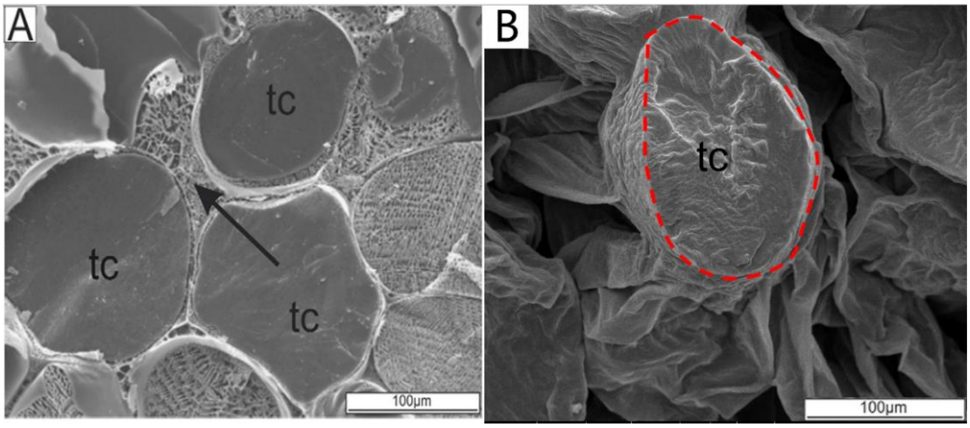

Figure S1: (A) Tannin cells in fresh persimmon slices (Ref. [31]), (B) Tannin cells after pressure-differential puffing drying.
